# High-Resolution, Motion-Corrected, Volume-Fused OCT for Investigating Longitudinal Changes in Subretinal Drusenoid Deposits in Intermediate AMD

**DOI:** 10.1167/tvst.14.6.15

**Published:** 2025-06-06

**Authors:** Jungeun Won, Antonio Yaghy, Stefan B. Ploner, Stephanie Kaiser, Jessica M. Girgis, Yunchan Hwang, Muhammad Usman Jamil, Andreas Maier, Nadia K. Waheed, James G. Fujimoto

**Affiliations:** 1 Department of Electrical Engineering and Computer Science, and Research Laboratory of Electronics, Massachusetts Institute of Technology, Cambridge, MA, USA; 2 New England Eye Center, School of Medicine, Tufts University, Boston, MA, USA; 3 Department of Computer Science, Pattern Recognition Lab, Friedrich-Alexander University Erlangen-Nürnberg (FAU), Erlangen, Germany

**Keywords:** optical coherence tomography, age-related macular degeneration, subretinal drusenoid deposits, motion correction, en face slab

## Abstract

**Purpose:**

To demonstrate high-resolution, motion-corrected, volume-fused optical coherence tomography (OCT) for assessing longitudinal changes in macular dot form subretinal drusenoid deposits (SDDs).

**Methods:**

Six consecutive isotropic volume raster scans over 6 × 6 mm (500 × 500 A-scans) were acquired using a high-resolution (2.7 µm axial resolution) spectral domain OCT prototype instrument. OCT volumes were computationally motion-corrected and fused. The distribution and longitudinal changes in dot SDDs were evaluated using en face OCT in a 50-µm-thick slab, from 27 µm above Bruch's membrane.

**Results:**

Computational motion correction and volume fusion methods improve visibility of small en face features and compensate for motion artifacts to facilitate longitudinal assessment. In total, 326 SDDs were identified in a representative series of four eyes from four patients with intermediate age-related macular degeneration (AMD) and assessed with a 3- to 12-month follow-up. Of the SDDs, 85.3% remained stable over the follow-up, while 9.8% regressed, 3.4% fused, and 1.5% new SDDs appeared.

**Conclusions:**

Computational motion correction and volume fusion combined with high-resolution OCT B-scans and en face slabs facilitate visualization and longitudinal tracking of focal pathologies, such as SDDs.

**Translational Relevance:**

The methods presented have the potential to improve OCT analysis of focal features, such as quantification of SDDs and other AMD biomarkers.

## Introduction

Age-related macular degeneration (AMD) is a leading cause of vision loss, significantly impacting the quality of life among the aging population.^1^ Subretinal drusenoid deposits (SDDs), also known as reticular pseudodrusen, are extracellular deposits that form between the retinal pigment epithelium (RPE) and the photoreceptors and were first described on fundus photography in 1990^2^ and on optical coherence tomography (OCT) in 2010.^3^ The prevalence of SDDs without AMD and with AMD is reported as 23% and 52%, respectively, with higher prevalence in intermediate AMD than early AMD (79% and 49%, respectively).^4^ SDDs have been recognized as an important risk factor for AMD progression,^5^ particularly for type 3 macular neovascularization and outer retinal atrophy.^6^^–^^8^ The dynamic nature of SDDs^9^—characterized by their potential to grow or regress—presents both a challenge and an opportunity for disease management and research investigation.^7^ Outer retinal atrophy following regression of dot SDDs and choroid thinning^10^^–^^12^ underscores the relevance for close monitoring of these deposits in AMD.

While SDDs are associated with the progression to late AMD,^6^^,^^11^^,^^13^ their small size, varying quantity, and diverse morphological patterns—ranging from dots and ribbons to midperipheral formations^11^—make accurate detection and classification challenging. Conventional imaging methods, such as color fundus photography, can miss a significant number of SDDs.^7^ Imaging methods, such as infrared reflectance imaging, infrared scanning laser ophthalmoscope, OCT, and fundus autofluorescence (FAF), are more sensitive for detecting SDDs. Advanced imaging techniques, such as adaptive optics scanning laser ophthalmoscopy (AOSLO), can provide detailed insights into the structure and behavior of SDDs and photoreceptors.^9^^,^^10^^,^^14^^,^^15^ However, the limited field of view of these advanced imaging techniques poses a barrier to their routine application in evaluating and tracking the progression of SDDs, as well as studying associated photoreceptor abnormalities.

OCT is advantageous for evaluating SDDs because cross-sectional B-scans unambiguously determine the subretinal location of these lesions, can be used for SDD staging (stages 1–3; determined based on the elevation of photoreceptor inner segment outer segment junction [IS/OS] or ellipsoid zone [EZ] and the decreased photoreceptor reflectivity),^11^ and provide information on outer retinal atrophy and type 3 neovascularization (OCT angiography). OCT is rapid, as well as more accessible and comfortable, compared to FAF and adaptive optics-based imaging methods. En face widefield OCT slabs have been shown as an effective method to detect SDDs compared to conventional imaging methods.^16^^–^^19^ A recent study also reported a substantial interreader agreement from six reading centers for evaluating SDDs based on OCT B-scans.^20^ However, the same study also noted only slight agreement for Stage 1 SDDs,^20^ potentially due to limited axial resolution and B-scan density. Substantial agreement was achieved on volume scans, compared to the fair agreement on selected B-scans.^20^ Thus, volume scans are critical to evaluate small SDDs and their topographic distribution in the macula, where dot SDDs are predominantly located in the inner ring and superior quadrant.^18^^,^^21^

In this study, we demonstrate high-resolution OCT and advanced motion correction and volume fusion technologies to generate dense, isotropic images that improve visualization of focal and low-contrast features, such as SDDs. Motion correction is a key component of volume fusion and facilitates longitudinal analysis of the dynamic changes of individual SDDs, as demonstrated in OCT en face slabs. This method can help elucidate the pathophysiological mechanisms underlying SDD dynamics and associated AMD progression and can be applied more generally to other focal features.

## Materials and Methods

### Subject Imaging

Patients with nonexudative intermediate AMD were recruited at the New England Eye Center at Tufts Medical Center (Boston, MA, USA) under protocols approved by the Institutional Review Boards at Tufts Medical Center and the Massachusetts Institute of Technology Committee for the Use of Humans as Experimental Participants. The procedures were compliant with the Declaration of Helsinki. Written informed consent was obtained from all patients before baseline imaging. The patients were longitudinally followed with an interval of at least 3 months and up to 12 months.

### High-Resolution OCT Prototype and Imaging Protocol

Imaging was performed with a high-resolution spectral domain OCT (SD-OCT) prototype instrument.^22^ The instrument operated at an 840-nm wavelength with an axial resolution of 2.7 µm, at a 128-kHz A-scan rate. Isotropic raster scans covering 6 × 6 mm (500 × 500 A-scans, 12-µm spacing, 2.4-second acquisition) were consecutively acquired in alternating orthogonal directions to collect a total of six OCT volumes in a single data acquisition of 14.6 seconds.

### Volume Fusion: Motion Correction, Illumination Correction, and Merging

Several challenges arise from acquiring volume data sets from older subjects, including eye motion and image distortion, variations in signal intensity, and frequent blinking. Our computational processing method compensates these effects and generates a single, consistent volume data set with improved signal to noise and without blurring from eye motion. Volume fusion performs computational motion correction, followed by illumination correction, residual artifact removals, flattening to the Bruch's membrane (BrM), and merging.

First, eye blinks were removed from the data set via thresholding. Next, eye motion and inconsistencies from optical image distortion were corrected. Detailed steps are described in our previously published study.^23^ In summary, we utilized A-scans, which are inherently motion artifact free in the axial direction, combined with minimally motion-distorted B-scans along the orthogonal transverse directions, which are rapidly scanned, to obtain image information along all three dimensions. A unique property of our computational method is that the eye motion within each volume raster scan was modeled as a continuous, time-dependent function to estimate specific three-dimensional displacements correcting eye motion for each A-scan. The fact that the raster scan directions were orthogonal and independent enabled motion estimation on the micron scale in both transverse and axial directions.^23^

Variations in signal intensity can affect image analysis and generate artifacts and therefore need to be compensated, particularly in elderly subjects. In individual volume raster-scanned images, this variation is less apparent and manifests as a low-frequency bias field because B-scans are acquired consecutively. However, when fusing multiple orthogonal volume raster scans, local A-scan acquisition time differences are much larger and can cause pronounced OCT signal intensity variation. Similar to the eye motion estimation, we modeled and estimated the illumination bias continuous along each B-scan. The continuity was then implicitly extended along the slow scan direction through the continuity along the orthogonal B-scans. Our constrained model guaranteed preservation of image features during illumination adjustment.^24^ The overall image brightness was kept consistent with the input data by locally adjusting the brightness of all volumes toward that of the brightest volume.

Next, machine learning–based segmentation was utilized to locate the BrM in each original B-scan.^25^ We motion-corrected the locations of BrM in each volume by applying the estimated displacements from computational motion correction and then averaged the depth location of BrM. To obtain a flattened OCT volume, we subtracted the average depth location of BrM from all A-scan locations and used the resulting displacement field to merge the motion-corrected image data. This volume was then used to generate en face slabs. Recent results demonstrate that our computation method enabled quantification of the RPE–Bruch's membrane features on a micrometer scale, across a wide age range of subjects and pathologies.^26^^,^^27^

### Grading of SDDs

An en face OCT slab with a thickness of 50 µm, located 27 to 77 µm above the BrM, was generated from the flattened and fused volumes. The en face OCT slabs at baseline and follow-up were registered between multiple visits and evaluated by two independent graders (A.Y. and J.W.). Open adjudication was performed by a third grader (S.K.). The presence or absence of SDDs was recorded and confirmed with the corresponding OCT B-scans. The number of SDDs and their quadrant location were documented at the baseline and follow-up visits. At follow-up, SDDs were categorized into four groups: new (if the SDD was not present at baseline but appeared at follow-up), regressed (if the SDD was present at baseline but disappeared at follow-up), fused (if the SDD fused with a nearby SDD at follow-up), or stable (if the SDD neither fused with a nearby SDD nor regressed at follow-up).

## Results

### Participants

A total of 326 SDDs were identified and tracked across four eyes from four patients with intermediate AMD over follow-up periods ranging from 3 to 12 months. The mean age of the participants was 75 ± 11 years. Participant demographics and clinical information are shown in the Table. The diagnoses for all eyes remained unchanged during the study period.

**Table.** Participants’ Summary

Diagnosis	Intermediate AMD			
Characteristic	Subject 1	Subject 2	Subject 3	Subject 4
Age, y	73	79	87	62
Sex	F	M	F	F
Eye	OD	OS	OD	OS
Refractive error, D	−0.50	−2.00	−1.50	−1.75
Follow-up interval. Mo	3	6	9	12
SDD presence	Yes	Yes	Yes	Yes

### Macular Dot Form SDDs Under En Face OCT

**Figure 1.**
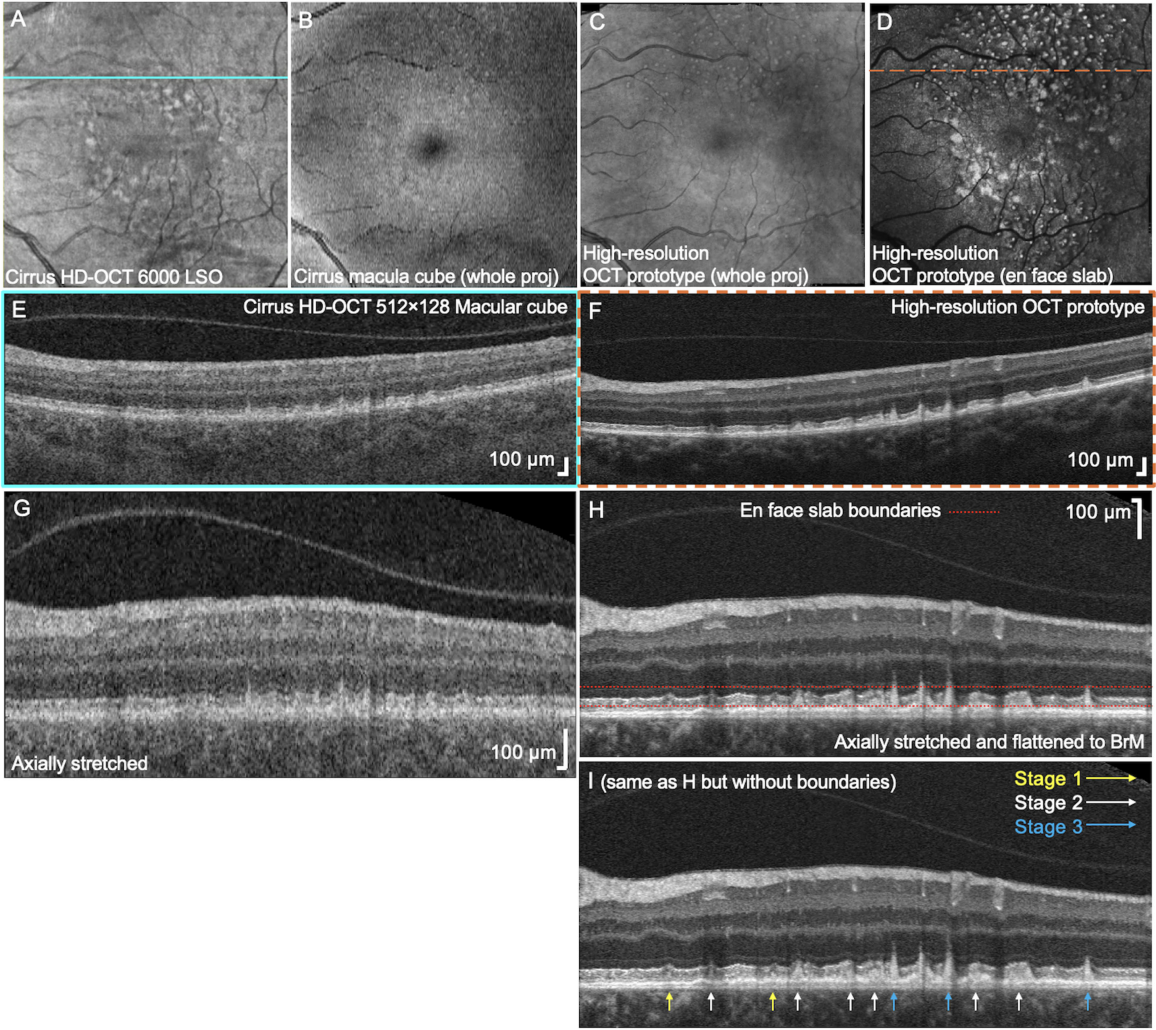
SDDs in an intermediate AMD eye (62-year-old woman). (**A**) Line scanning ophthalmoscope (LSO) image on a Cirrus HD-OCT 6000 is shown. The *light blue line* indicates OCT scan region (E, G). (**B**) En face whole projection of 512 × 128 macular cube protocol on a Cirrus HD-OCT 6000. (**C**) En face whole projection of 500 × 500 (6 mm × 6 mm) motion-corrected and volume-fused macular cube acquired using a high-resolution SD-OCT prototype instrument. (**D**) An averaged en face slab projected from 27 to 77 µm above Bruch's membrane highlights dot and ribbon forms of SDDs. The *dotted orange line* indicates OCT scan region (F, H, I). (**E**) Corresponding OCT B-scan at the *light blue line* (A) acquired from a Cirrus HD-OCT macular cube. (**F**) Corresponding OCT B-scan at the *dotted orange line* (D) acquired using the high-resolution OCT prototype. (**G**) The same OCT B-scan (E) is axially stretched and flattened. (**H**, **I**) The same OCT B-scans (F) are axially stretched and flattened to improve visualization of the slab positions (*red dotted lines*) and indicate various stages of the SDDs. Although they are the same OCT B-scans, SDDs are better visualized in panels H and I than in F.

Figure 1 compares OCT macular cube scans acquired using the Cirrus HD-OCT 6000 (Carl Zeiss Meditec, Dublin, CA, USA) (B-scan spacing of 47 µm), one of the standard OCT imaging protocols in clinical practice, versus motion-corrected and volume-fused high-resolution OCT prototype data (B-scan spacing of 12 µm). A 50-µm-thick slab from 27 to 77 µm above the BrM was chosen to detect earlier stage of SDDs accumulating on the RPE. SDDs aggregating near the RPE (labeled as stages 1 to 3, Fig. 1I) are clearly revealed in the corresponding high-resolution OCT B-scan and captured within the en face slab boundaries shown by the red dotted lines (Fig. 1H). The OCT B-scans were axially stretched to highlight the deposits of Stage 1 SDDs (Figs. 1H. 1I). A corresponding B-scan acquired from Cirrus HD-OCT was also compared (Figs. 1E, 1G), which highlights the superior axial resolution of our prototype and the importance of flattening and axial stretching for improving the visibility of the SDDs.

**Figure 2.**
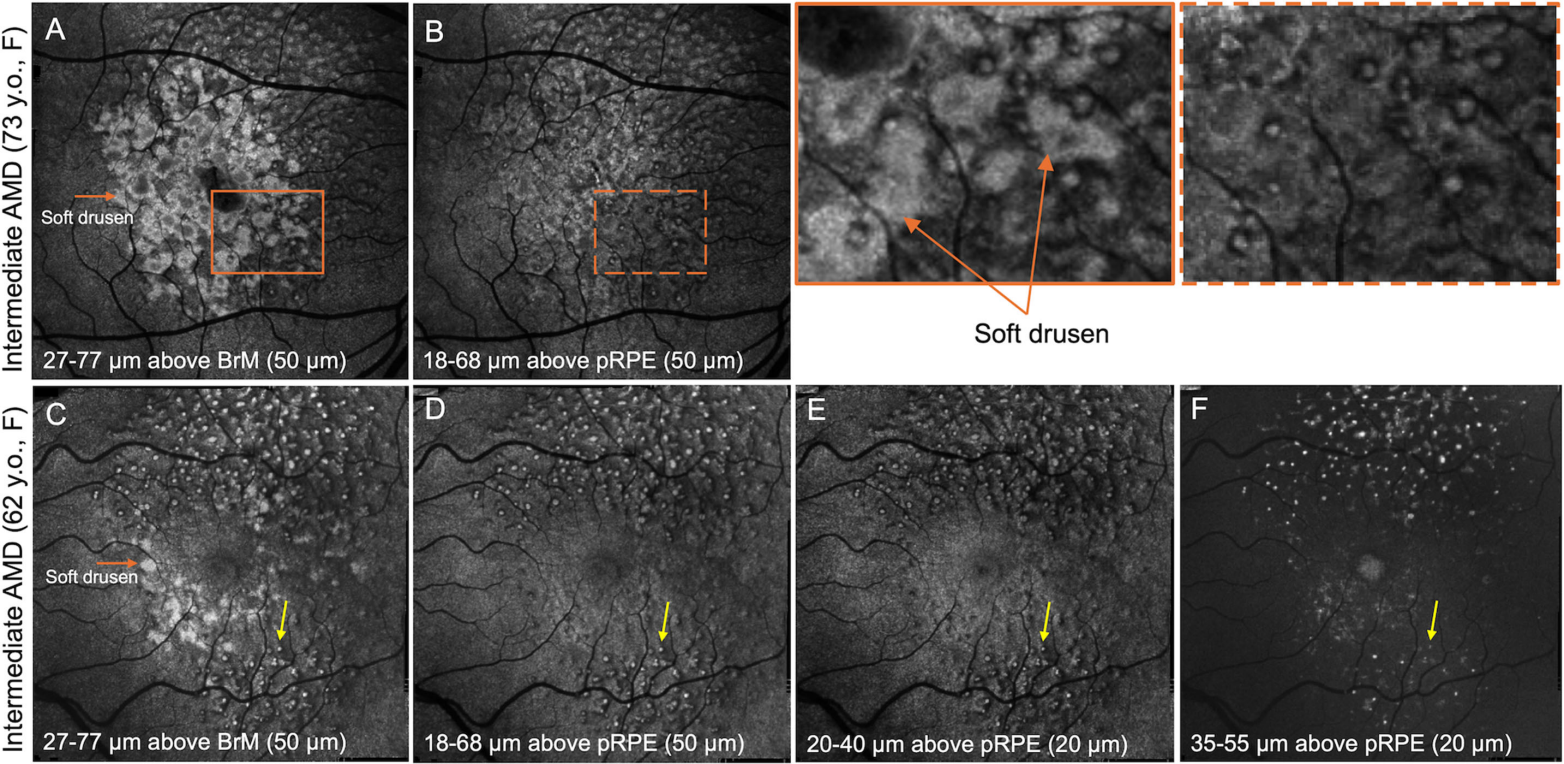
Comparison of different OCT en face slab generation methods (6 × 6 mm). (**A**, **B**) A 50-µm-thick en face slab generated from BrM and posterior RPE (pRPE), from a female subject (73 years old) diagnosed with intermediate AMD. The slab generated from BrM also reveals the presence of soft drusen (*orange arrow*). (**C**, **D**) A 50-µm-thick en face slab generated from BrM and pRPE from a female subject (62 years old) diagnosed with intermediate AMD. The slab generated from BrM also reveals the presence of soft drusen (*orange arrow*). (**C–F**) Different slab thickness and positions highlight differences in visualizing SDDs (*yellow arrow*).

Figure 2 shows en face OCT slabs generated using different thicknesses and positions. If the en face slab is generated from the posterior boundary of the RPE rather than BrM, large soft drusen will not appear in the en face slab, which may improve the visualization of SDDs. However, simultaneous observation of the SDDs and soft drusen in AMD may facilitate interpretation of the en face OCT slabs in clinical practice, and thus, we have chosen the slab generated from the BrM.

### Advanced Computational Methods Remove Distortions and Improve Visibility of SDDs

**Figure 3.**
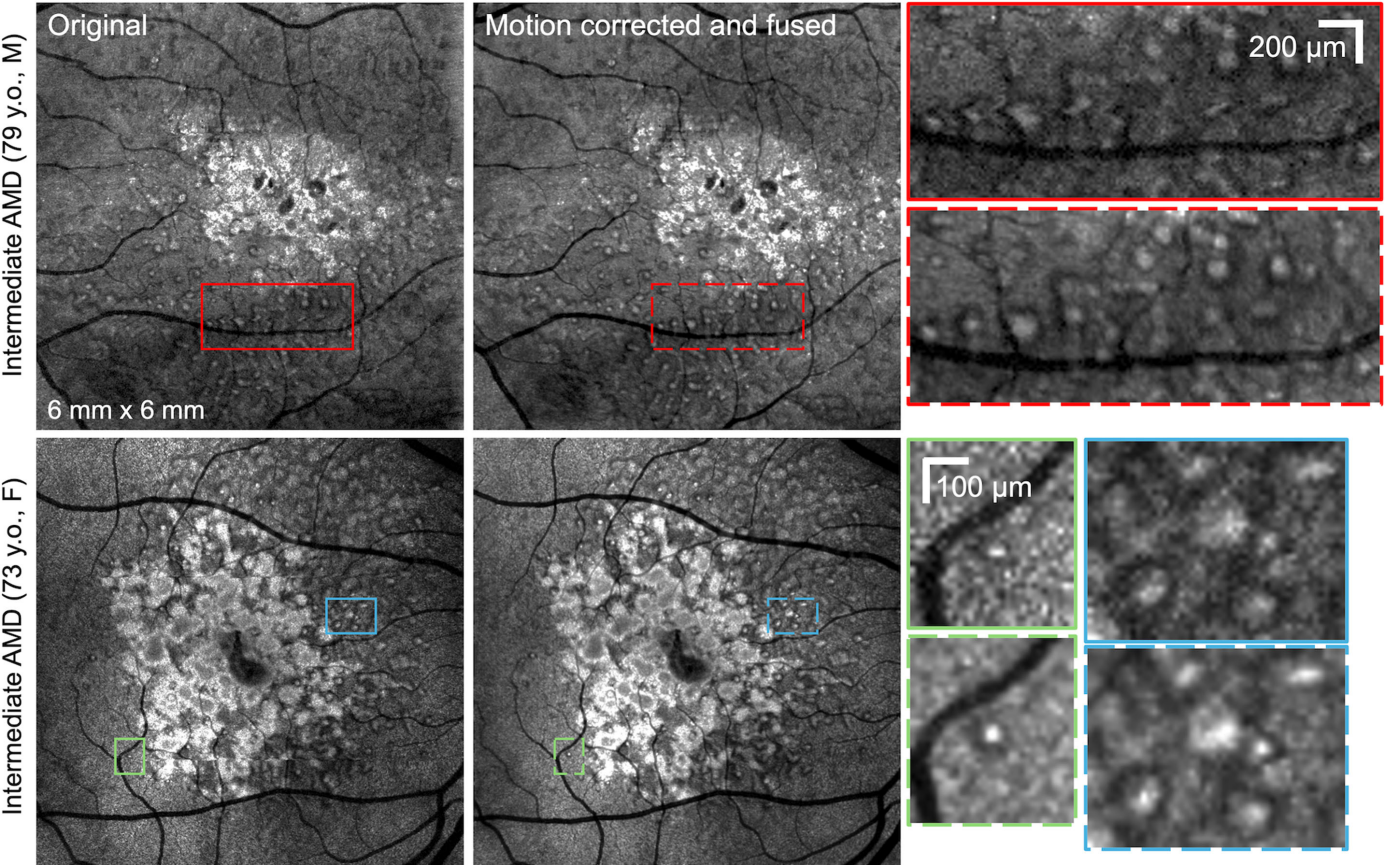
Three-dimensional motion correction and volume fusion methods improve the visibility of macular dot form SDDs and correct distortions, enabling accurate assessment and longitudinal comparison. OCT en face slab from 27 to 77 µm above the BrM from original high-resolution OCT versus motion-corrected volume-fused OCT. Zoomed-in regions show the improvement of SDD visualization from motion correction and volume fusion by correcting displacements from eye drift and saccades and improving signal-to-noise ratio.

**Figure 4.**
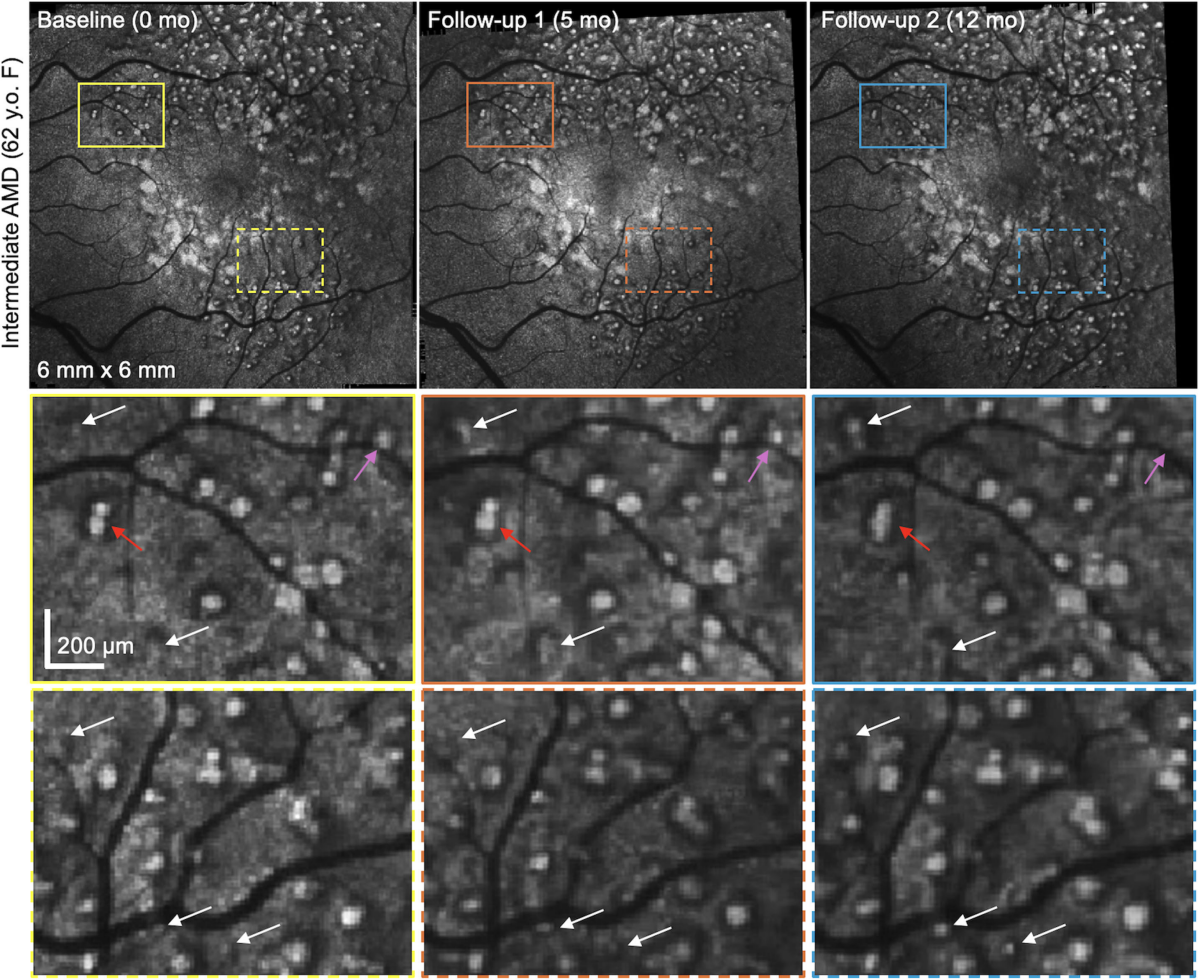
Longitudinal changes of SDDs evaluated using en face OCT slabs. Over a follow-up period of 12 months, this intermediate AMD eye showed fusion, newly appearing, regressed, and stable SDDs in the en face OCT slabs. Zoomed-in images are shown in the bottom two *rows*. *White arrows* indicate newly appearing, *red arrows* indicate fused SDDs, and *pink arrows* indicate regressed SDDs.

Computational motion correction and volume fusion methods improve the visibility of dot SDDs and facilitate longitudinal assessment. The volume-fused image in Figure 3 shows an improvement in contrast of the dot SDDs. Compared to a single-volume scan, the artifacts from saccadic eye movements are removed (red boxes). Drift eye motion correction is particularly beneficial for longitudinal assessment where images acquired at different time points are compared (Fig. 4), by ensuring accurate overlap of corresponding structures. Because even minor motion artifacts from saccades and drift eye motions can distort the appearance of small dot SDDs, accurate motion correction methods are essential for characterizing SDDs and examining their longitudinal changes in presence, shape, and size.

### Longitudinal Changes of Dot SDDs

Figure 4 shows representative longitudinal changes in an intermediate AMD eye, where the morphologic changes suggest merging and regression of SDDs over a follow-up period of 12 months. Dynamic changes of individual dot SDDs are indicated by different-colored arrows in the zoomed-in region. White, pink, and red arrows denote changes corresponding to new, regressed, and fused SDDs, respectively. An intermediate AMD eye with a shorter 3-month follow-up did not show any noticeable changes in SDDs (not shown).

**Figure 5.**
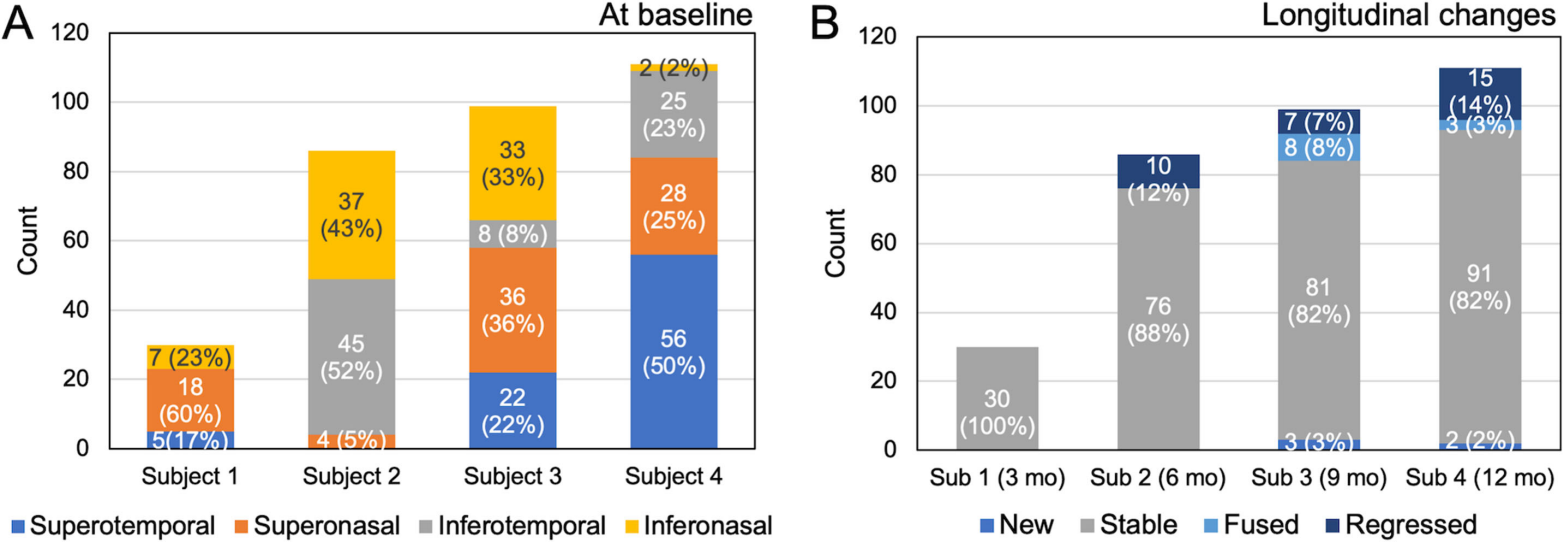
Spatial distributions of SDDs at baseline and their longitudinal changes are summarized for each subject. (**A**) At baseline, SDDs were located dominantly in the superior region, except one eye (subject 2). (**B**) Reported changes for each eye indicate most SSDs remained stable, and some changes were observed at a follow-up visit longer than 3 months.

Over the follow-up period, most SDDs (85.3%, *n* = 278) remained stable. However, a notable proportion of SDDs underwent changes. Figure 5 summarizes the distributions and changes of SDDs: 9.8% (*n* = 32) of SDDs regressed and were no longer detectable at follow-up, 3.4% (*n* = 11) of SDDs fused with nearby SDDs, and 1.5% (*n* = 5) of new SDDs were identified, which were not present at baseline. The distribution of SDDs across retinal quadrants was relatively uniform and stable, with 26.4% (*n* = 86) in the superonasal quadrant, 25.5% (*n* = 83) in the superotemporal quadrant, 24.2% (*n* = 79) in the inferonasal quadrant, and 23.9% (*n* = 78) in the inferotemporal quadrant at the baseline.

## Discussion

Accurate volumetric motion correction and fusion methods are essential for correcting eye motion distortions and visualizing small focal features, such as dot SDDs, as well as enabling longitudinal studies. Using our volume fusion approach and isotropic volume scans of high-resolution OCT, individual SDDs across 6-mm × 6-mm fields were assessed using a single, multivolume acquisition. The high-resolution OCT prototype used in this study does not employ hardware eye tracking–based motion correction, which is more common in commercial OCT instruments but instead performs computational motion correction after acquiring multiple consecutive volumes in a single acquisition. Commercial high-resolution instruments can also acquire isotropic, dense volume raster scans similar to those used in this study. However, the accuracy of commercial eye-tracking systems is limited by the accuracy and latency of eye motion detection. In addition, eye tracking does not compensate for axial eye motion. Instead, retrospective processing must be applied. It is possible to apply volumetric motion correction and/or volume fusion methods in commercial instruments if manufacturers implement the scan protocols and enable export of appropriate data formats (raw OCT data with higher bit depth). Motion correction and volume fusion methods will become increasingly important as finer-scale structural biomarkers of disease progression are developed.

Utilizing the en face OCT slab for assessment has the advantage that it does not require segmentation of SDDs. Prior work using en face OCT slabs has shown their utility and effectiveness to detect Stage 2 and 3 SDDs, and multiple thicknesses and ranges of the slab have been suggested.^16^^–^^19^ For example, one group utilized a 20-µm-thick slab from 35 to 55 µm above the RPE on a prototype SS-OCT instrument (Carl Zeiss Meditec, Dublin, CA, USA),^16^^,^^17^ whereas another group utilized a 40-µm-thick slab from 48 to 88 µm above the BrM on an Optovue Solix SD-OCT instrument (Visionix, Fremont, CA, USA).^18^ One study demonstrated multiple en face slabs (inner slab anterior to the IS/OS [EZ] and outer slab on the RPE) to detect and differentiate types of drusen, including SDDs, cuticular drusen, and soft drusen.^19^ In the current study, we chose a sufficient slab thickness to include the region anterior to the RPE as well as the region anterior to the IS/OS (EZ) in order to have higher contrast from varying-stage SDDs. However, the optimal slab position and thickness may depend on pathologies (i.e., presence of thick basal laminar deposits, sub-RPE fluid), system specifications, and scan protocols.

The capability of tracking individual dot SDDs can potentially provide valuable insights into their changes and distribution patterns in the macula. In the literature, the growth of deposits coincides with the progressive shortening of overlying photoreceptors.^28^^–^^30^ As the disease advances, Müller glial cells have been hypothesized to clear the deposits through the retina, which was observed during the progression to outer retinal atrophy.^28^

A study published in 2020 by Zhang et al.^29^ investigated the dynamic nature of SDDs using a combination of AOSLO and SD-OCT. The study tracked the progression of individual SDDs over a 12-month period in a cohort of six eyes from four patients with AMD. Their findings revealed a spectrum of SDD evolution, with 69% of SDDs exhibiting growth, 15% undergoing shrinkage, and 6% remaining stable in size. Notably, 11% of the SDDs completely disappeared during the study, while a small fraction (0.6%) demonstrated a unique pattern of regression followed by reemergence.^29^ In the current study, while most SDDs (85.3%) remained stable in presence over the follow-up period, a notable proportion underwent changes, including regression (9.8%), fusion (3.4%), and new formation (1.5%). Nonetheless, it is important to note that our classification of “stable” SDDs does not preclude potential growth or subtle changes in size.

OCT can provide a 6-mm × 6-mm (or larger) macular field of view. In this study, the quadrant analysis of SDD distribution revealed a relatively uniform distribution across the retina, but with a predominance in the superior region in three of four AMD eyes. As recently reviewed,^31^ the distributions of SDDs and soft drusen in AMD have been shown to follow the distributions of cone and rod photoreceptors on the outer retina. Future studies investigating spatial variations in SDD dynamics across quadrants will be important to determine topographical differences in the formation and clearance of these deposits.

Several limitations of our study should be acknowledged. The study has a small sample size, which limits generalizability to the broader AMD population. Larger studies with longer, consistent follow-up intervals are needed to study the spectrum of SDD dynamics and its natural history. Only macular dot form SDDs were assessed, and SDDs located outside the 6-mm × 6-mm field of view could not be detected.

Despite these limitations, our study demonstrates the utility of high-resolution, motion-corrected, volume-fused OCT and en face OCT for visualizing and longitudinally assessing SDDs. This computational method opens new avenues for investigating dynamic changes and the pathophysiology of SDDs, as well as other focal pathologies in AMD. Future studies with large enrollment and longer and more standardized follow-up are warranted. Developing automated detection and measurement methods would be important for analyzing these larger data sets. Furthermore, correlating SDD dynamics with functional outcomes, such as microperimetry and rod-mediated dark adaptation, may yield valuable prognostic indicators for AMD progression.
